# Protein Associations With Alcohol Consumption and Genetic Risk for Alcohol‐Related Sociomedical Conditions

**DOI:** 10.1111/adb.70045

**Published:** 2025-06-25

**Authors:** Gabin Drouard, Teemu Palviainen, Chia‐Ling Kuo, Breno S. Diniz, Xiaoling Wang, Miina Ollikainen, Antti Latvala, Jaakko Kaprio

**Affiliations:** ^1^ Institute for Molecular Medicine Finland (FIMM) HiLIFE, University of Helsinki Helsinki Finland; ^2^ Department of Public Health Sciences University of Connecticut Health Center Farmington Connecticut USA; ^3^ The Cato T. Laurencin Institute for Regenerative Engineering University of Connecticut Health Center Farmington Connecticut USA; ^4^ UConn Center on Aging University of Connecticut Health Center Farmington Connecticut USA; ^5^ Department of Clinical and Biomedical Sciences University of Exeter Exeter UK; ^6^ Georgia Prevention Institute, Medical College of Georgia Augusta University Augusta Georgia USA; ^7^ Minerva Foundation Institute for Medical Research Helsinki Finland; ^8^ Institute of Criminology and Legal Policy University of Helsinki Helsinki Finland

**Keywords:** alcohol consumption, genetics, major depressive disorder, polygenic risk scores, proteomics, substance use, twins

## Abstract

Studies investigating proteomic associations with alcohol consumption and the genetic links of these proteins to alcohol‐related traits are scarce. The aims of our study were (1) to identify proteins associated with alcohol consumption and (2) to investigate the molecular pathways and genetics linking the identified proteins to alcohol consumption and related sociomedical conditions. We generated proteomic and genotypic data from blood samples of 387 Finnish twins (age range: 56–70) and calculated polygenic risk scores (PRSs) of eight alcohol‐related traits: obesity, alcohol dependence, number of drinks per week, number of cigarettes per day, major depressive disorders (MDDs), schizophrenia, externalising behaviour and educational attainment. We identified 20 (out of 2321) proteins associated with alcohol consumption, expressed as log ethanol grams per month, after Bonferroni correction and adjustment for BMI, sex and age. Within‐pair analyses in monozygotic twin pairs showed that some of the identified associations persisted after accounting for genetic confounding. While only the PRS representing genetic risk for the number of alcoholic drinks per week was associated with alcohol consumption, several proteins were associated with PRSs, in particular the PRS of MDD. All identified proteins were significantly replicated in the UK Biobank, and pathway analysis suggested their collective connection to alcohol consumption might be explained by oxidative stress and cell damage. In conclusion, we identified several alcohol‐associated plasma proteins whose levels are also linked to genetic risk for mental illness and substance use. Our study suggests the potential of proteins as biomarkers for early detection of alcohol‐related disorders.

## Graphical abstract

This study identified blood proteins associated with alcohol consumption in Finnish twins, with all findings replicated in the UK Biobank. Using polygenic risk scores and twin models, we found that some proteins shared genetics with major depressive disorder, while others remained associated with alcohol consumption independently of genetic effects. These insights may aid biomarker discovery.

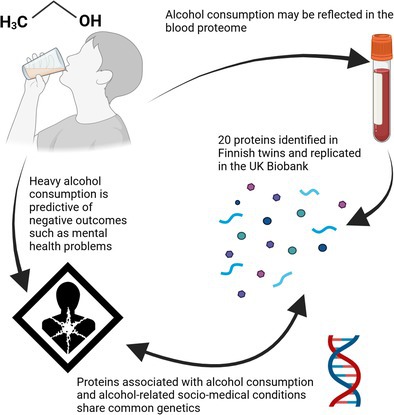

## 1 Introduction

Heavy alcohol consumption is a major public health concern as it causes or contributes to the development of multiple somatic diseases including liver cirrhosis, cancers and cardiovascular disease and is also closely associated with many mental health problems [1, 2, 3]. In particular, high alcohol consumption increases the risk of major depression [4]. Individuals diagnosed with schizophrenia are at greater risk for high alcohol consumption as well [5]. Heavy drinking in midlife has also been shown to be associated with cognitive decline and dementia in old age [6]. Alcohol consumption also correlates with other traits: heavy drinking positively associates with weight gain and higher body mass index (BMI) [7, 8]. Externalising behaviour problems [9] and lower educational attainment [10] are also known to be positively associated with subsequent alcohol use disorders and increased alcohol consumption. Overall, the effects of alcohol consumption and its frequency and patterns of use on disease risk and behaviour are complex, involving both genetic and environmental factors. The use of so‐called omics data, whether representing underlying DNA sequence (genotypes) or the end products of gene function (proteins), may pave the way to a better understanding of the molecular features of the public health burden of alcohol use and inform new treatment strategies for alcohol‐related disorders.

The use of omics data has already led to great success in identifying the effect of genes on alcohol consumption and alcohol‐related conditions. Genome‐wide association studies (GWAS) of alcohol consumption and alcohol use disorders have also identified a large number of SNPs in genes associated with alcohol consumption [11, 12]. For example, GWAS have identified hundreds of SNPs in genes associated with the development of depression and schizophrenia [13, 14]. Identified SNPs can then be used to create polygenic risk scores (PRSs), whose use in disease stratification or clinical practice has shown promise in the study of common and complex traits [15].

Proteomics is one of the most promising omics for gaining biological insight into disease or predicting disease survival, as proteins are the gene end product, reflect genome functionality and are more proximal to the phenotypes of interest [16]. A few proteomic studies have already identified blood plasma proteins associated with diseases that correlate with alcohol consumption, such as alcohol‐related liver disease [17], liver function or depressive episodes [18]. However, studies investigating the blood proteomic associations of alcohol consumption are less common, although such knowledge would allow earlier detection of effects of alcohol on disease predisposition, especially since alcohol consumption is likely a contributing cause of the identified associations with protein levels. The largest blood proteomic analysis to date is that of the UK Biobank (UKB), in which proteomic associations of smoking, but not alcohol consumption, have been performed [18]. Another large‐scale study examined the associations of plasma proteins with more than 300 traits, including alcohol dependence but not alcohol frequency or consumption [16]. However, one study investigated blood proteomic associations with several traits, most of them related to cardiovascular disease, including alcohol consumption [19]. The authors of this study showed that several proteins overlap across traits and diseases, suggesting that the blood plasma proteome is a rich resource for linking alcohol consumption to other conditions. Another study by Corlin et al. identified 30 plasma proteins out of 1305 proteins tested that were associated with alcohol consumption (grams/day) [20]. The same study identified associations between proteins and SNPs associated with, for example, chronic pancreatitis, gout and metabolic syndrome, thereby improving genetic knowledge of how these proteins are genetically related to cardiometabolic disease. However, whether proteins associated with alcohol consumption are associated with genetic risks for traits other than cardiometabolic ones, such as those related to education, mental health and behaviour, seems to remain unexplored.

Our study is aimed at contributing to the literature by (1) identifying plasma proteins associated with alcohol consumption and (2) investigating the molecular pathways and genetics linking the identified proteins to alcohol consumption and related sociomedical conditions. To address Aim 2, we (i) performed pathway analysis on the identified set of proteins, (ii) assessed associations between levels of the identified proteins with PRSs for up to eight traits related to alcohol consumption and (iii) used twin study designs. We used a sample of nearly 400 Finnish twins from which Olink proteomic data (> 2000 proteins identified) and genotype data were quantified. Finally, we replicated the identified associations between proteins and alcohol consumption in the UKB.

## 2 Material and Methods

### 2.1 Participants and Data Collection

The current study is based on twins from the older Finnish Twin Cohort who have been enrolled in a follow‐up substudy: the Essential Hypertension Epigenetics (EH‐Epi) study [21]. Twin pairs discordant for blood pressure were initially selected to participate in this study [22]. The twins were invited to an in‐person assessment to measure their blood pressure, weight and height, to complete questionnaires and interviews and to provide venous blood samples after an overnight fast [23]. From the questionnaires, alcohol consumption was assessed and estimated as ethanol grams per month (ETOH g/month) as described elsewhere [24]. These measurements have been used in previous investigations and have shown relatively strong predictive ability for mortality [25] and alcohol‐related causes of death [26] and covaried longitudinally with weight gain [27]. The ETOH g/month were log‐transformed in the current study to reduce skewness in the variable (skewness was 4.3 before the log transformation and −1.3 after). As some participants were nondrinkers, we used a modified log transformation *f:x ↦ log(1 + x)* so that for nondrinkers *f(0)* is defined and equals to zero. Thus, all individuals had positive log‐transformed alcohol consumption. BMI at blood sampling, age at blood sampling and sex were used as covariates in the analyses, with BMI defined as the ratio of weight (kilogramme) to the square of height (metre). Genotype and proteomic data were generated from the blood samples as described below. A description of the EH‐Epi sample is provided in Table 1.

**TABLE 1:** Description of the EH‐Epi twins with proteomic data. IQR, interquartile range; *N*, number of twins for which information is available or for which a particular binary trait is present; SD, standard deviation.

		Available information (N)	Mean or N (%)	SD	IQR	Range
Continuous variables	Alcohol consumption (g/months)	387	321	429.9	70.2–385.5	0.0–4928.0
	Alcohol consumption (log g/month)	387	5.2	1.6	4.3–6.0	0.0–8.5
	Body mass index (kg·m^2^)	401	27.3	4.9	24.0–29.6	18.1–46.1
	Waist circumference (cm)	401	94.4	14.6	84.5–103.0	56.0–140.0
	Waist‐to‐height ratio	401	0.56	0.08	0.51–0.61	0.37–0.89
	Systolic blood pressure (mmHg)	401	143.3	16.9	131.5–154.5	104.2–217.5
	Diastolic blood pressure (mmHg)	401	83.5	10.1	77.2–89.5	58.5–123.2
Binary variables	Females	401	237 (59)			
	Never smokers	398	188 (47)			
	Former smokers	398	146 (36)			
	Daily smokers	398	53 (13)			
	Depression diagnosis	391	63 (16)			
	Antidepressant medication	401	29 (7)			

### 2.2 Proteomics Data Processing

Proteomic profiling was performed on plasma samples from 415 EH‐Epi participants, and data processing was performed as described elsewhere [28]. Briefly, samples were analysed using an antibody‐based technology (Olink Proteomics AB, Uppsala, Sweden), and proteomic data were generated using the Olink Explore 3072 platform which includes Explore 384 cardiometabolic (I + II), inflammation (I + II), neurology (I + II) and oncology (I + II) panels. Proteins for which values below the limit of detection (LoD) exceeded 20% were excluded. Values below the LoD for the remaining proteins, representing less than 1% of the total data points, were replaced with the plate‐specific LoD values. Detection of outlier samples and internal quality control were performed [28], resulting in a final dataset of 2321 proteins quantified in 401 individuals. Of these individuals, 387 had complete information about their alcohol consumption. Protein values are expressed as Normalised Protein eXpression (NPX) values, which is Olink's unit for quantifying relative protein concentrations.

### 2.3 Polygenic Risk Scores

Genotype data were generated using Illumina and Affymetrix arrays [29]. For the 387 EH‐Epi twins with proteomic and alcohol consumption data, 378 had also genotype data available. We calculated eight PRSs from publicly available GWAS describing genetic susceptibility to (i) obesity (number of SNPs used: *N*_SNP_ = 996 919) [30], (ii) alcohol dependence (*N*_SNP_ = 1 135 412) [11], number of (iii) drinks per week (*N*_SNP_ = 1 143 064) or (iv) cigarettes per day (*N*_SNP_ = 1 143 620) [12], (v) major depressive disorder (MDD) (*N*_SNP_ = 1 147 810) [31], (vi) schizophrenia (*N*_SNP_ = 1 144 587) [32], (vii) externalising behaviour (*N*_SNP_ = 1 020 738) [33] and (viii) educational attainment (*N*_SNP_ = 1 145 339) [34]. Data processing of these PRSs has been described elsewhere [29]. We corrected each of the PRSs for population stratification by regressing out the top 10 genetic principal components [35] and scaled the residuals to a mean of zero and unit variance. Scaling the residuals allowed for better interpretation of the results, since each unit of scaled PRS represents a one standard deviation (SD) change in polygenic risk for the above PRSs.

### 2.4 Statistical Analyses

We quantified associations between alcohol consumption and plasma proteins using linear mixed‐effects models. We modelled alcohol consumption as an independent variable and included BMI, sex and age at blood sampling as covariates. Proteins were *z*‐scored and used as dependent variables (i.e., as outcomes). To correct for correlation within the data due to familial relationship between the cotwins, we added a family identifier as a random effect. We tested for null associations and corrected the resulting *p* values using Bonferroni to correct for multiple testing, resulting in a *p* value threshold of *p* = 0.05/2321 = 2.2e−5 for significance. In addition, we tested downstream interactions of sex with alcohol consumption in models by testing nullity of the interaction terms between sex and alcohol consumption.

Once we identified proteins associated with alcohol consumption, we sought to explore the molecular determinants of these associations in a three‐step process. First, we used the Reactome pathway database [36] to investigate the molecular pathways to which the identified proteins belonged. We provided the UniProt IDs of the identified proteins as input to the Reactome database. Then, we tested associations between the identified proteins and alcohol consumption with PRSs for alcohol‐related traits, using PRSs as independent variables with sex, BMI and age at blood sampling as covariates. Multiple testing correction with Bonferroni was performed to correct for the number of tests performed between each PRS with identified proteins. Finally, because our sample included 107 complete pairs of monozygotic twins with proteomic and alcohol data, we used within‐pair twin designs to assess whether the identified associations between plasma proteins and alcohol consumption persisted while correcting for genetic confounding [37]. To do this, we fitted linear regressions modelling differences in protein levels (as dependent variable) with differences in alcohol consumption (as independent variable) within a pair. Difference in BMI between cotwins was used as a covariate. Because the twins came to the on‐site visit for blood samples at the same time as their cotwin, differences in age at blood sampling were not included as a covariate and MZ twins are always matched on sex. Nullity of coefficients was tested, and nominal *p* values were corrected using Bonferroni correction.

### 2.5 Replication in the UKB

We aimed to replicate associations between proteins and alcohol consumption identified in EH‐Epi twins in the UKB. Of the active participants in the UKB baseline cohort, 53 014 were included in the UK Biobank Pharma Proteomics Project (UKB‐PPP). The mean age of this subset was 56.8 years (range: 39–70), with a mean BMI of 27.5. The majority of participants were White (93.7%), followed by Black (2.3%), Asian (2.3%) and Other (1.7%). The cohort comprised 53.9% females. Regarding smoking status, 54% were never smokers, 35% were former smokers and 11% were current smokers.

Protein expression was measured using the Olink Explore 3072 platform and normalised to account for technical variability. Alcohol intake frequency (UKB field ID 1558) was assessed at recruitment by the touchscreen question: ‘About how often do you drink alcohol?’ The response (*n* = 52 889) followed the distribution below: never (8.7%), special occasions only (11.8%), one to three times a month (10.9%), once or twice a week (25.9%), three or four times a week (22.5%), daily or almost daily (20.2%). In analyses, these responses were assigned scores approximating the number of days per year of alcohol consumption, 0, 6, 24, 78 (=1.5 × 52), 182 (=3.5 × 52) and 365, respectively.

Each protein, standardised to *z*‐scores within the UKB‐PPP cohort, was tested for association with frequency of alcohol consumption using linear regression models with proteins as dependent variables. Models were adjusted for age, sex, ethnicity, BMI and smoking status. The mean change in protein levels per unit increase in alcohol consumption was reported, along with the standard error and *p* value. To account for multiple testing (number of tests: 20), *p* values were adjusted using Bonferroni's method. All hypothesis tests were two‐sided, and adjusted *p* values below 0.05 were considered statistically significant. Statistical analyses were conducted using R Version 4.1.1.

## 3 Results

### 3.1 Study Participant Characteristics in the EH‐Epi Sample

A description of the EH‐Epi twins with available proteomic data is provided in Table 1. The mean reported alcohol consumption in the sample of 387 twins having proteomic data was 321 ETOH g/month (SD: 430 g/month). Once log‐transformed, the mean log alcohol consumption was 5.2 log ETOH g/month (SD = 1.6). Participants' age at blood sampling ranged 56–70 years and averaged 62.3 years.

### 3.2 Proteomic Analysis Reveals 20 Proteins Associated With Alcohol Consumption

We quantified associations between alcohol consumption and plasma proteins using repeated linear mixed‐effects models adjusted for BMI, sex and age. A total of 20 proteins were significantly associated with alcohol consumption after Bonferroni correction (Table 2), with carboxypeptidase A1 showing the highest level of significance. Altogether 13 of the 20 proteins were positively associated with alcohol consumption. In interaction analyses designed to assess potential sex interactions with alcohol consumption, nominal *p* values below 0.05 for interaction terms were identified only for two proteins, thyroglobulin (*p* = 0.04) and chymotrypsinogen B (*p* = 0.05), indicating that these two associations may be stronger in men. However, this evidence disappeared when correcting for multiple testing.

**TABLE 2:** Significant associations between alcohol consumption and Olink plasma proteins in the EH‐Epi sample, all replicated in the UKB. Only associations with Bonferroni‐corrected *p* values indicating a nonnull association between proteins and alcohol consumption (*p* < 0.05) in the EH‐Epi sample are shown. All proteins were subjected to replication analyses in the UKB, using frequency of alcohol consumption as an independent variable. The names of the proteins are given along with their UniProt IDs and coding genes. se, standard error.

			Results in the EH‐Epi sample (discovery)				Results in the UKB (replication)		
Protein description					p values			p values	
Name	UniProt	Gene	Estimate	se	Nominal	Bonferroni	T value	Nominal	Bonferroni
Carboxypeptidase A1	P15085	CPA1	0.19	0.03	5.5E−10	1.3E−06	23.9	8.2E−126	7.4E−125
Trypsin‐2	P07478	PRSS2	0.19	0.03	5.7E−10	1.3E−06	22.6	9.6E−113	6.7E−112
Apical endosomal glycoprotein	Q6UXC1	MAMDC4	0.19	0.03	8.9E−10	2.1E−06	71.6	9.9E−324	2.0E−322
Carcinoembryonic antigen‐related cell adhesion molecule 16	Q2WEN9	CEACAM16	−0.18	0.03	2.3E−09	5.4E−06	−61.6	9.9E−324	2.0E−322
Carboxypeptidase B	P15086	CPB1	0.17	0.03	2.1E−08	5.0E−05	18.5	3.7E−76	2.2E−75
Chymotrypsinogen B	P17538	CTRB1	0.15	0.03	2.4E−07	5.5E−04	7.2	7.6E−13	7.6E−13
C4b‐binding protein beta chain	P20851	C4BPB	−0.16	0.03	4.7E−07	1.1E−03	−52.8	9.9E−324	2.0E−322
Oxytocin‐neurophysin 1	P01178	OXT	0.14	0.03	2.6E−06	6.0E−03	43.0	9.9E−324	2.0E−322
Adhesion G‐protein‐coupled receptor D1	Q6QNK2	ADGRD1	0.14	0.03	3.8E−06	8.8E−03	36.0	4.2E−279	4.7E−278
Beta‐Ala‐His dipeptidase	Q96KN2	CNDP1	0.14	0.03	4.2E−06	9.8E−03	37.8	1.8E−308	2.3E−307
Sushi domain‐containing protein 5	O60279	SUSD5	−0.14	0.03	5.1E−06	1.2E−02	−26.4	2.2E−152	2.2E−151
Chymotrypsin‐like elastase family member 2A	P08217	CELA2A	0.13	0.03	6.6E−06	1.5E−02	17.3	1.6E−66	8.1E−66
Argininosuccinate synthase	P00966	ASS1	0.14	0.03	6.6E−06	1.5E−02	17.0	8.4E−65	3.4E−64
Thyroglobulin	P01266	TG	−0.14	0.03	7.7E−06	1.8E−02	−14.9	3.7E−50	1.1E−49
Receptor‐type tyrosine‐protein phosphatase S	Q13332	PTPRS	−0.14	0.03	8.6E−06	2.0E−02	−44.4	9.9E−324	2.0E−322
B‐cell antigen receptor complex‐associated protein beta chain	P40259	CD79B	−0.14	0.03	9.5E−06	2.2E−02	−38.5	6.2E−320	8.7E−319
Glutathione hydrolase 1 proenzyme	P19440	GGT1	0.13	0.03	9.9E−06	2.3E−02	37.2	1.8E−299	2.1E−298
Heparan‐sulphate 6‐O‐sulfotransferase 2	Q96MM7	HS6ST2	−0.13	0.03	1.0E−05	2.4E−02	−23.2	6.1E−118	4.9E−117
Serpin I2	O75830	SERPINI2	0.12	0.03	1.5E−05	3.4E−02	10.3	1.2E−24	2.3E−24
CD166 antigen	Q13740	ALCAM	−0.13	0.03	1.7E−05	3.9E−02	−43.8	9.9E−324	2.0E−322

### 3.3 Replication in the UKB

All 20 proteins that we identified in the EH‐Epi sample were significantly associated with frequency of alcohol consumption in the UKB (Table 2), with high degree of evidence, since the UKB‐PPP sample is larger than that of the EH‐Epi. All coefficients in EH‐Epi were of the same sign as the *t* values reported in the UKB, suggesting that the associations between alcohol consumption and protein levels were also consistent in direction.

### 3.4 Pathway Analysis Highlights Key Biological Pathways Enriched Among the Identified Proteins

We performed pathway analysis using the Reactome database for the set of 20 identified proteins. Five biological pathways were identified with FDR‐corrected *p* values below 0.05: ‘developmental lineage of pancreatic acinar cells’, ‘developmental cell lineages’, ‘activation of matrix metalloproteinases’, ‘defective GGT1 causes GLUTH’ and ‘defective GGT1 in aflatoxin detoxification causes GLUTH’. Pathway outputs from the Reactome database are available in the supporting information (Table S1).

### 3.5 Within‐Pair Analyses in Monozygotic Twin Pairs Suggest That Associations Are Not Driven by Genetic Factors Alone

We used within‐pair twin designs in monozygotic twin pairs to assess whether identified associations between plasma proteins and alcohol consumption persisted after correction for genetic confounding (Table 3). Altogether 11 of the 20 identified associations had nominal *p* values below 0.05, indicating that genetics is unlikely the only driver of these associations. The associations between alcohol consumption with carcinoembryonic antigen‐related cell adhesion molecule 16 and heparan‐sulphate 6‐O‐sulfotransferase 2 proteins remained significant after correction of the nominal *p* values for multiple testing (Table 3).

**TABLE 3:** Within‐pair analyses in monozygotic twin pairs assess the robustness of identified associations to correction for genetic confounding. Significant associations identified between plasma proteins and alcohol consumption in the EH‐Epi sample were examined in within‐pair analyses to assess whether they would persist after correction for genetic confounding. Within‐pair analyses were conducted in 107 monozygotic twin pairs and consisted of fitting linear regressions modelling differences in alcohol consumption with differences in protein levels, while adjusting for differences in BMI. Multiple testing correction was performed using the Bonferroni method (20 tests). Ten of the 20 associations had nominal *p* values below 0.05, with only one association having a *p* value following Bonferroni correction below 0.05 which involved the carcinoembryonic antigen‐related cell adhesion molecule 16. UniProt IDs and coding genes of the investigated proteins are listed in Table 2.

Protein	Estimate	se	Nominal p	Bonferroni p
Carcinoembryonic antigen‐related cell adhesion molecule 16	−0.19	0.05	2.3E−04	4.6E−03
Heparan‐sulphate 6‐O‐sulfotransferase 2	−0.18	0.05	4.8E−04	9.6E−03
Thyroglobulin	−0.14	0.05	6.0E−03	1.2E−01
Sushi domain‐containing protein 5	−0.14	0.05	6.2E−03	1.2E−01
Carboxypeptidase A1	0.14	0.05	7.2E−03	1.4E−01
C4b‐binding protein beta chain	−0.13	0.05	1.1E−02	2.3E−01
B‐cell antigen receptor complex‐associated protein beta chain	−0.12	0.05	1.4E−02	2.9E−01
Argininosuccinate synthase	0.12	0.05	1.8E−02	3.7E−01
Chymotrypsin‐like elastase family member 2A	0.12	0.05	2.0E−02	4.0E−01
Receptor‐type tyrosine‐protein phosphatase S	−0.11	0.05	3.7E−02	7.4E−01
Carboxypeptidase B	0.10	0.05	4.6E−02	9.1E−01
Serpin I2	0.10	0.05	5.3E−02	1.0E+00
Trypsin‐2	0.10	0.05	5.8E−02	1.0E+00
Glutathione hydrolase 1 proenzyme	0.08	0.05	8.3E−02	1.0E+00
CD166 antigen	−0.08	0.05	9.7E−02	1.0E+00
Beta‐Ala‐His dipeptidase	0.09	0.05	1.0E−01	1.0E+00
Adhesion G‐protein‐coupled receptor D1	0.06	0.05	1.9E−01	1.0E+00
Oxytocin‐neurophysin 1	0.06	0.05	2.2E−01	1.0E+00
Apical endosomal glycoprotein	0.05	0.05	3.4E−01	1.0E+00
Chymotrypsinogen B	0.04	0.05	4.6E−01	1.0E+00

### 3.6 Only the PRS for Number of Alcoholic Drinks per Week Is Associated With Alcohol Consumption

We sought to investigate whether alcohol consumption is associated with genetic risk for up to eight sociomedical conditions related to alcohol consumption in the EH‐Epi sample. We observed a significant positive association between alcohol consumption and higher genetic risk to drink alcohol, the latter depicting the genetically determined number of alcoholic drinks per week (estimate: 0.18, *p* = 0.04). No significant associations were found between alcohol consumption and genetic susceptibility to obesity (estimate: 0.00, *p* = 0.99), alcohol dependence (estimate: 0.10, *p* = 0.26), number of cigarettes per day (estimate: −0.01, *p* = 0.87), MDD (estimate: −0.07, *p* = 0.39), schizophrenia (estimate: 0.05, *p* = 0.59), externalising behaviour (estimate: 0.11, *p* = 0.21) and educational attainment (estimate: −0.09, *p* = 0.30). Thus, only the PRS for the number of alcoholic drinks per week was associated with alcohol consumption.

### 3.7 Identified Proteins Are Linked to Genetic Risk for Diseases and Behaviours Correlated With Alcohol Use

**FIGURE 1:**
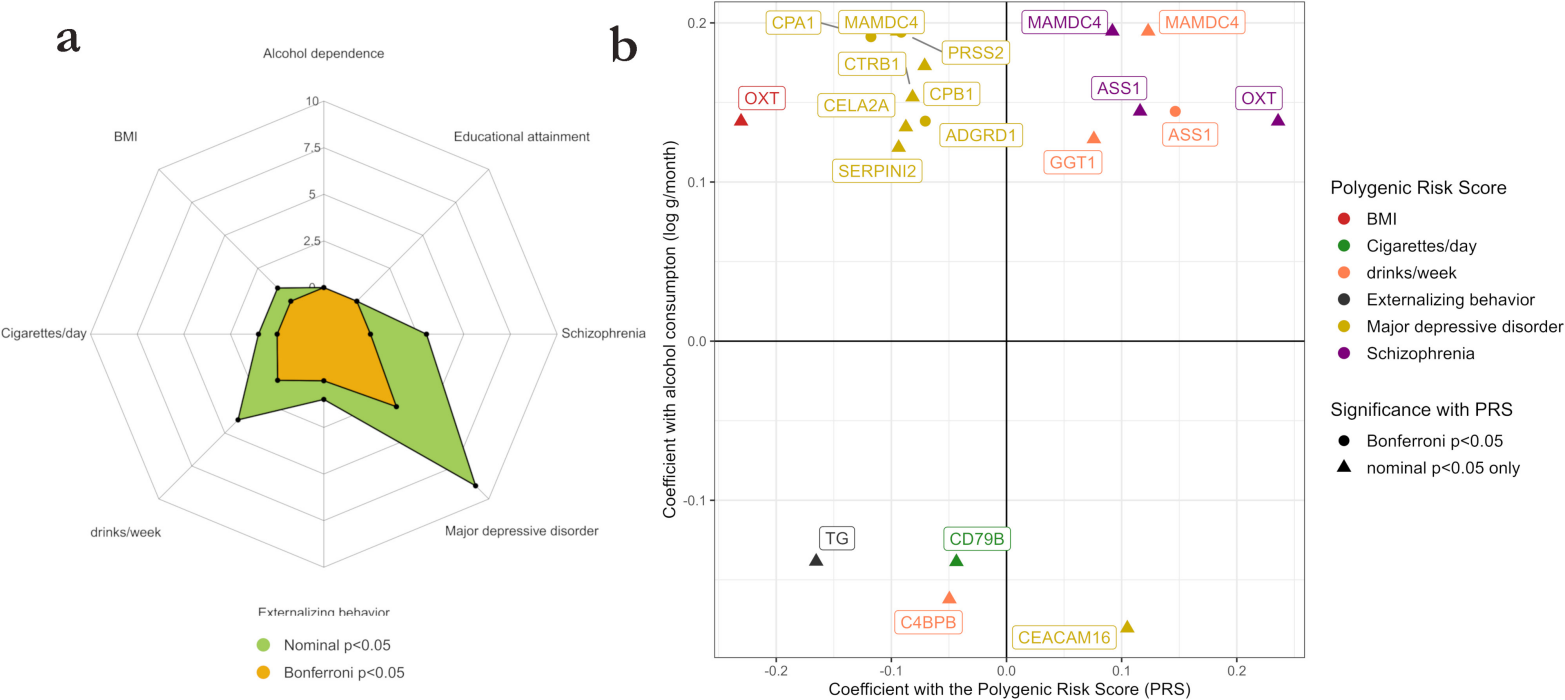
Associations between identified proteins and polygenic risk scores in the EH‐Epi sample. (a) Radar plot showing the number of associations with nominal (green zone) or Bonferroni‐corrected (yellow zone) *p* values below 0.05. Summary statistics are available in the supporting information (Table S1). (b) Scatterplot of coefficients between proteins with alcohol consumption (*y*‐axis) and a polygenic risk score (PRS) (*x*‐axis) for PRS–protein associations with nominal *p* values below 0.05. Associations that do not pass Bonferroni correction are represented with triangle shapes. Summary statistics are available in the supporting information (Table S1).

We examined associations between the identified proteins and PRSs for the eight sociomedical conditions correlated with alcohol consumption (Figure 1). We observed 19 associations between protein levels and PRSs with nominal *p* values below 0.05. Of these, 9 were with the PRS for MDD and 4 with the PRS for alcoholic drinks per week. The PRSs for alcohol dependence and educational attainment showed no association with protein levels with a nominal *p* value below 0.05. After correcting for multiple testing for each PRS (*p* value threshold following Bonferroni correction: *p* = 0.05/20 = 2.5e−3), 4 associations remained significant, 3 of which were with the PRS for MDD. Proteins associated with the PRS for MDD were trypsin‐2 (coding gene: *PRSS2*), carboxypeptidase A1 (*CPA1*) and adhesion G‐protein‐coupled receptor D1 (*ADGRD1*). The fourth association was between argininosuccinate synthase (*ASS1*) and the PRS for alcoholic drinks per week. Summary statistics are available in the supporting information (Table S2).

## 4 Discussion

Our study identified 20 proteins associated with alcohol consumption in nearly 400 Finnish adult participants. Additional analyses showed that the set of proteins we identified (1) are rooted in defined biological pathways (which we discuss later), (2) may share genetics with behavioural and psychiatric traits, particularly MDD, and (3) are not associated with alcohol consumption solely due to genetic factors. Thus, our study provides insight into the links between alcohol consumption, related proteins and alcohol‐related diseases by demonstrating that alcohol‐associated proteins are intrinsically and genetically linked to the predisposition to develop traits and disorders correlated with alcohol consumption. Ultimately, the proteins we identified could offer valuable insights into how alcohol consumption might contribute to disease onset and may serve as candidate biomarkers, potentially linking even to long‐term conditions such as dementia or cognitive decline.

Among the proteins we identified to associate with alcohol consumption, some have been previously reported to associate with alcohol or alcohol‐related diseases. For example, trypsin‐2 has been positively associated with alcohol consumption in another study [20]. We also identified the protein chymotrypsin‐like elastase family member 2A, which is related to elastase and therefore related to pancreatic secretion of trypsin. Another protein we identified is serpin I2, a member of the serpin family that has been implicated in alcohol‐related liver disease [17]. Another protein associated with alcohol consumption is oxytocin‐neurophysin 1, whose role is to transport oxytocin, a hormone involved in the reproductive system and known to be associated with addictive behaviour [38]. Therefore, a few of the identified proteins are relevant biomarker candidates to be investigated for early identification of individuals at risk for alcohol‐related diseases. As for carcinoembryonic antigen‐related cell adhesion molecule 16 (*CEACAM16*), despite reported associations with liver disease and function in the literature [39], this protein does not appear to have been previously reported to be associated with alcohol consumption. Our study indicates that higher alcohol consumption is associated with lower *CEACAM16* levels and that this association persists in within‐pair analyses of monozygotic twin pairs, suggesting that environmental factors influence this association.

Pathway analysis revealed five biological pathways, grouped into three main families, related to the set of proteins we identified. The first family of pathways we identified involves developmental cell lineages. This pathway may be relevant to the disruption of pancreatic acinar cells, which are known to be affected by heavy alcohol consumption [40, 41]. The second family of pathways we identified is the matrix metalloproteinase pathway; matrix metalloproteinases are important enzymes involved in ECM degradation. The metalloproteinase pathway is associated with tissue remodelling processes that are very active after tissue damage, including in the liver [42]. Animal models have shown increased levels of metalloproteinases after long‐term alcohol consumption due to oxidative stress [43]. The third pathway family was that of defective GGT1 pathways, which have been shown in the literature to be associated with heavy alcohol consumption and related diseases [44, 45, 46]. Gamma‐glutamyl transferases (GGT) regulate intracellular glutathione (an antioxidant) levels to protect cells from oxidative stress that can occur during metabolism, such as alcohol consumption. High levels of plasma GGT may be a sign of liver disease induced by alcohol. It is commonly used as a biomarker of alcohol use, though it is not specific to alcohol exposure. Thus, pathway analysis suggests that the set of proteins we identified may be related to alcohol consumption because of the cell damage and oxidative stress induced by alcohol consumption. However, the evidence for the involvement of these pathways in the observed associations between proteins and alcohol consumption remains limited, as only a handful of proteins have been identified in relation to these pathways.

We observed multiple associations between the levels of the identified proteins and PRSs of alcohol‐related traits. Most of these associations were with the PRS of MDD, three of which remained significant after Bonferroni correction and involved the proteins: trypsin‐2, carboxypeptidase A1 and adhesion G‐protein‐coupled receptor D1. The association of trypsin‐2 with depressive symptoms has been reported in a study using proteomic data from the UKB [18], and adhesion G‐protein‐coupled receptors have been associated with psychiatric disorders in the literature [47]; the role of carboxypeptidase A1 in depression remains unclear. Associations between these plasma proteins and the PRS of MDD were negative, while being positively associated with alcohol consumption. A recent study found a negative association between a PRS of MDD and alcohol consumption in males [48], and this association tended to be negative in our sample as well, without reaching statistical significance. The direction of the associations between these proteins, alcohol consumption and genetic risk for MDD is therefore consistent with a scenario in which individuals at higher genetic risk for MDD have lower levels of proteins and alcohol consumption, with the latter likely causally influencing the former. Further studies are needed to shed light on why individuals with a higher genetic predisposition to MDD have lower alcohol consumption and through which biological processes, especially since depression and alcohol consumption are mostly reported to be positively associated in the literature [2]. A recent transcriptome‐wide association study found a negative association between a gene encoding a serine protease (*PRSS16*), which is in the same protein family as trypsin‐2, and MDD [49], but we found no additional evidence to explain negative associations between plasma proteins and PRS of MDD. Associations are likely bidirectional and may be confounded by other effects, genetic or environmental. Biomarker analyses may help to resolve the complex relationship between alcohol use, alcohol use disorders and mood disorders.

Limitations of our study are mainly related to the low statistical power, especially due to the relatively modest size of the twin sample we investigated. In addition, the number of proteins tested was relatively large (number of proteins: 2321), which led to a stringent correction for multiple testing with Bonferroni and *p* values below 2.2e−5 to indicate statistical significance. Therefore, the number of proteins we identified as associated with alcohol consumption is likely to be underestimated. However, despite the limited number of proteins identified, replication analyses in the UKB suggest that these proteins are serious biomarker candidates for alcohol use, as they all replicated significantly. Lack of statistical power was also likely reflected in the within‐pair analyses, as only 2 in 11 associations with a nominal *p* value below 0.05 passed multiple testing correction (20 tests). Another challenge in research on alcohol use is the assessment of alcohol use itself, which in the current study was based on questionnaires. These measures can be affected by various biases (e.g., recall bias) and depend on the population and time period studied. However, the same measures of alcohol use have been used in the cohort earlier and are shown to be highly predictive of alcohol‐associated causes of death (see supporting information in [26]). Thus, the replicability of molecular findings of alcohol (mis‐)use to other populations is challenging. However, the replication of our findings in the UKB and the biological pathways underlying the protein signature we identified suggest our results to be relatively robust. Finally, although the EH‐Epi sample is a selected sample recruiting twin pairs previously identified as discordant for blood pressure, the alcohol consumption and health profiles (Table 1) of the twins are comparable to those of their age groups in the general population. Thus, the results of this study are likely to be well generalisable to the general population, as we have also demonstrated with replication in the UKB.

In conclusion, our study identified several alcohol consumption–associated plasma proteins whose role in the literature is partially established and whose role as early biomarkers of disease deserves further investigation. Pathway analysis suggests that identified proteins may associate with alcohol consumption through cell damage and excessive oxidative stress. We additionally provide evidence that proteins associated with alcohol consumption share genetics with MDD, shedding light on the complex processes by which alcohol consumption may translate into diseases and related disabilities.

## Author Contributions

G.D. and J.K. designed the study. X.W., M.O. and J.K. were involved in data collection. Proteomic data processing and statistical analysis in EH‐Epi were performed by G.D. T.P. generated the polygenic risk scores for EH‐Epi twins. C.‐L.K. and B.S.D. replicated protein associations in the UK Biobank. G.D. drafted the initial version of the manuscript. All authors participated in the interpretation of the results, reviewed and revised the original draft of the manuscript and approved the final version.

## Ethics Statement

The study protocol was approved by the Institutional Ethics Board of the Hospital District of Helsinki and Uusimaa, Finland (ID 154/13/03/00/11), and the Institutional Review Board of Augusta University. All applicable written and informed consent was obtained in relation to the data generated or used for analysis.

## Conflicts of Interest

The authors declare no conflicts of interest.

## Supporting information


**Table S1.** Associations between identified proteins and polygenic risk scores with nominal *p* values less than 0.05. PRS: polygenic risk score. se: standard error.

**Table S2.** Supporting information.

## Data Availability

The data that support the findings of this study are available on request from the corresponding author. The data are not publicly available due to privacy or ethical restrictions.
